# Default from tuberculosis treatment in Tashkent, Uzbekistan; Who are these defaulters and why do they default?

**DOI:** 10.1186/1471-2334-8-97

**Published:** 2008-07-22

**Authors:** Epco Hasker, Maksad Khodjikhanov, Shakhnoz Usarova, Umid Asamidinov, Umida Yuldashova, Marieke J van der Werf, Gulnoz Uzakova, Jaap Veen

**Affiliations:** 1 Project HOPE Tuberculosis Control Program for the Central Asia Region, Almaty, Kazakhstan; 2 KNCV Tuberculosis Foundation, The Hague, The Netherlands; 3 Department of Infectious Diseases, Tropical Medicine & AIDS, Center for Infection and Immunity Amsterdam (CINIMA), Academic Medical Center, University of Amsterdam, The Netherlands; 4 Project Implementation Unit GFATM, Uzbekistan; 5 Epidemiology and Disease Control Unit, Department of Public Healt, ITM, Antwerp, Belgium

## Abstract

**Background:**

In Tashkent (Uzbekistan), TB treatment is provided in accordance with the DOTS strategy. Of 1087 pulmonary TB patients started on treatment in 2005, 228 (21%) defaulted. This study investigates who the defaulters in Tashkent are, when they default and why they default.

**Methods:**

We reviewed the records of 126 defaulters (cases) and 132 controls and collected information on time of default, demographic factors, social factors, potential risk factors for default, characteristics of treatment and recorded reasons for default.

**Results:**

Unemployment, being a pensioner, alcoholism and homelessness were associated with default. Patients defaulted mostly during the intensive phase, while they were hospitalized (61%), or just before they were to start the continuation phase (26%). Reasons for default listed in the records were various, 'Refusal of further treatment' (27%) and 'Violation of hospital rules' (18%) were most frequently recorded. One third of the recorded defaulters did not really default but continued treatment under 'non-DOTS' conditions.

**Conclusion:**

Whereas patient factors such as unemployment, being a pensioner, alcoholism and homelessness play a role, there are also system factors that need to be addressed to reduce default. Such system factors include the obligatory admission in TB hospitals and the inadequately organized transition from hospitalized to ambulatory treatment.

## Background

Uzbekistan is a country in Central Asia with a population of just over 26 million. With the collapse of the Soviet Union in 1991, Uzbekistan became an independent country. It inherited the Soviet system of tuberculosis (TB) control, which is a highly vertical system. The system relies on active case finding and on individualized case management by specialist doctors in in-patient facilities. Though reasonably effective, the system was very expensive. When resources became scarce in the mid 1990s, it could no longer be maintained and tuberculosis spiralled out of control. Case notification rates more than doubled between 1995 and 2005 [1]. The problems were further compounded by the emergence of multi drug resistant TB (MDR-TB). A recent survey shows a 14.8% MDR-TB prevalence among new TB patients in the Uzbek capital Tashkent [2]. The WHO estimate for HIV prevalence among new TB cases in Uzbekistan for 2006 was still less than 1% [1].

In search of a more cost effective system, Uzbekistan embarked upon a phased implementation of the DOTS strategy, 100% DOTS coverage was achieved in 2005 [3]. Under the new strategy, case finding is mostly passive and there has been a shift towards ambulatory treatment. Patients are still hospitalized during the intensive phase but continuation phase treatment is provided on an out-patient basis, usually at a general primary health care (PHC) facility. Treatment regimens have been standardized; the regimen for new patients consists of a 2 months intensive phase with Isoniazid, Rifampicin, Pyrazinamide and Ethambutol, followed by a 4 month continuation phase with Rifampicin and Isoniazid [4]. However treatment is under the control of specialist doctors and duration of both phases of treatment is often extended based on radiographic findings.

Overall the new approach to TB control has been successful; like in other former Soviet Union settings [5,6] there has been improvement in some of the main epidemiological indicators. For Uzbekistan treatment success rates prior to DOTS implementation are not available, but the TB mortality rate went down from 12.5 per 100,000 population in 2002 to 8.5 per 100,000 in 2006. The overall treatment success rate for new smear positive cases registered during 2005 in Uzbekistan was 81% (72% cured, 9% treatment completed).

For the capital city Tashkent, which has a population of 2.3 million, the treatment success rate was considerably lower. Only 58% of 394 new smear positives registered during 2005 were treated successfully (52% cured, 6% treatment completed). The largest single cause for unsuccessful treatment was 'defaulter' which is defined as 'A patient who interrupted treatment for two months or more' [4]. Of all new smear positive TB patients registered in Tashkent during 2005, 18% defaulted. When considering all 1087 pulmonary TB cases (smear positives as well as smear negatives) registered in Tashkent during 2005, the defaulter rate was even higher with 21% (228 patients) defaulting. Other causes of poor treatment outcome among smear positives in Tashkent were 'Failure' (10%), 'Died' (9%) and 'Transferred out' (5%). The overall defaulter rate among new smear positives for Uzbekistan was 7%, which is comparable to defaulter rates in the surrounding Central Asian countries [1].

The defaulter rate in Tashkent is far above average. Various studies in Africa, Asia and Latin America have shown different reasons for default, the main reasons being lack of time to regularly visit a health facility[7,8], lack of money for transport [7], poverty, lack of material incentives[9] and most of all lack of information or insufficient health education [7,8,10,11]. In countries of the former Soviet Union, different circumstances apply. Recent studies from Russia identified substance abuse, unemployment and homelessness as important risk factors for default [12-15]. In the perception of TB facility staff in Tashkent, much of the default is the result of patients moving abroad in search of employment opportunities. In this study, we investigated risk factors for default and time of default. We also collected information on the recorded reasons for default.

## Methods

We used a case control approach. The study population consisted of new pulmonary TB patients registered in Tashkent City during 2005. A new patient was defined as a patient who at the time of registration had never taken treatment for tuberculosis, or had taken treatment for less than one month [4]. Records of TB patients who defaulted in 2005 (cases) and records of patients who did complete treatment (controls) were reviewed. We enrolled adult pulmonary TB patients, aged 18 years and above.

We calculated the sample size based on the assumption that unemployment is an important risk factor for default, as it leads to migration in search of labour. In an earlier study, we found that 26% of TB patients in Uzbekistan were unemployed [16]. Assuming an odds ratio of 2.5, a sample size of 120 cases and 120 controls is required to demonstrate with 95% certainty and with a power of 90% that unemployment is a statistically significant risk factor for default. Since we found no apparent seasonal pattern in default we decided to study a cohort of defaulters started on treatment since the 1^st ^of January, 2005. Taking into account the possibility of missing records, we included in our sample all 153 defaulters started on treatment during the first half of 2005 and an equal number of controls. Controls were randomly sampled, using the information in the Electronic Surveillance and Case Management (ESCM) database, among the new pulmonary TB patients that started treatment in Tashkent in the same period as the sample cases but did complete treatment.

Standardized data collection forms were developed and pre-tested. Data collection was conducted by staff members of Project HOPE, an international non governmental organization providing technical assistance to the national TB program of Uzbekistan. Information was obtained primarily from reviewing TB-01 patient record cards (the standard WHO TB patient record card) and patient files kept at the TB dispensaries. When required, clarification was requested and obtained from TB specialists who had been the treating physicians. Thus data was collected on various factors that might be associated with default:

• Demographic factors: sex and age

• Social factors: marital status and employment status

• Risk factors for default: concomitant disease, HIV infection, history of imprisonment, homelessness, unemployment, migration, alcohol abuse and injecting drug use

• TB treatment records: date of start of treatment, initial sputum smear result, and date of last drug intake.

• Current TB treatment status

In addition, we collected information for the defaulters about the duration of treatment and type of health facility providing treatment at the time of default and reasons for default.

Data were entered in Microsoft Access in duplicate by two different persons and the two files were compared to identify typing errors. For data analysis we used Stata/SE V9.0 (Stata Corp., College Station Tx, USA). Observed associations were assessed through Chi Square tests and bivariate and mulitivariate analysis. All variables with a p-value <0.25 in bivariate analysis were included in the multivariate logistic regression model. Variables for the final model were selected using backward elimination, based on the probability of the likelihood-ratio statistic based on the maximum partial likelihood estimates. The probability of removal was set at p = 0.10.

The study was approved by the National Research Institute for Pulmonology and Phtisiatry in Tashkent.

## Results

We were able to retrieve medical records of 144 defaulters (94%) and of all 153 selected controls. When reviewing the paper records, it turned out that there were discrepancies between treatment outcomes recorded in the district TB register and those entered in the ESCM database. The district TB register is the main document used by TB control program staff, we therefore used it as our final reference. In the defaulters group we included only those listed in this register as 'defaulters', and in the control group we included only those listed in this register as 'cured' or 'treatment completed'. As a result 18 (12.5%) out of 144 cases and 21 (13.7%) out of 153 controls had to be excluded from the analysis. Records of the remaining 258 patients, 126 defaulters and 132 controls, were included in the analysis.

The study population consisted of 174 men (67%) and 84 women (33%); ages ranged from 18 to 83 years, the median age was 37 years. Most study subjects were married (54%), and unemployment was common (40%). For further details we refer to table 1.

**Table 1:** Characteristics of the study population and risk factors for default.

**Variable**	**Cases**	**Controls**	**Bivariate analysis**	**Multivariate analysis**
	**n (%)**	**n (%)**	**OR (95% CI)**	**OR (95% CI)**

Sex				
Male	89 (71)	85 (64)	1.33 (0.79–2.25)	
Female	37 (29)	47 (36)	1.0*	

Age groups				
18–24	19 (15)	30 (23)	1.0*	
25–34	33 (26)	31 (24)	1.63 (0.76–3.48)	
35–44	30 (24)	27 (20)	1.75 (0.81–3.81)	
45–54	23 (18)	27 (20)	1.35 (0.60–2.99)	
55–64	12 (10)	10 (8)	1.89 (0.69–5.24)	
65+	9 (7)	7 (5)	2.03 (0.65–6.36)	

Civil state				
Married	64 (51)	74 (56)	1,0*	
Single	35 (28)	37 (28)	1.09 (0.62 – 1.94)	
Widowed	8 (6)	5 (4)	1.85 (0.58 – 5.94)	
Divorced	13 (10)	7 (5)	2.15 (0.81 – 5.71)	

Occupation				
Employed by government	15 (12)	31 (24)	1.0*	1.0*
Employed in private sector	7 (6)	6 (5)	2.41 (0.69 – 8.44)	2.63 (0.73 – 9.57)
Self-employed	10 (8)	13 (10)	1.59 (0.57 – 4.45)	1.74 (0.60 – 5.09)
Unemployed	56 (44)	46 (35)	2.52 (1,21 – 5.22)	2.73 (1.28 – 5.86)
Pensioner	22 (18)	15 (11)	3.03 (1.23 – 7.46)	4.07 (1.57 – 10.52)
Pupil or student	4 (3)	10 (8)	0.83 (0.22 – 3.07)	0.98 (0.25 – 3.73)
House wife	11 (9)	11 (8)	2.07 (0.73 – 5.84)	2.15 (0.73 – 6.31)

Risk factors for Default				
HIV infection	6 (5)	8 (6)	0.78 (0.26 – 2.30)	
Other concomitant disease	28 (22)	32 (24)	0.89 (0.50 – 1.59)	
History of imprisonment	12 (10)	5 (4)	2.67 (0.91 – 7.82)	
Homelessness	6 (5)	0	n.a.	
Alcohol abuse	17 (14)	4 (3)	4.99 (1.63 – 15.28)	6.01 (1.68 – 19.47)
Migrant	1 (1)	0	n.a.	
Intravenous drug user	2 (2)	2 (2)	1.05 (0.15 – 7.56)	

Type of pulmonary TB				
Smear negative	94(75)	80 (61)		
Smear positive	32 (25)	52 (39)	0.57 (0.33 – 0.97)	0.42 (0.24 – 0.75)

* Used as reference

From the records it was possible to assess how long patients had been on treatment for 117 (93%) defaulters and 130 (98%) controls. The median duration of treatment was 69 days for defaulters (IQR 50–92 days) and 200 days for controls (IQR 187–230 days). Although treatment regimens have been standardized, duration of treatment is often extended at the discretion of the treating TB specialists. Seventy one defaulters (61%) defaulted during the intensive phase after a median treatment duration of 51 days; 15 (13%) defaulted during the continuation phase after a median treatment duration of 114 days. The remaining 31 (26%) completed the intensive phase but did not start the continuation phase, their median treatment duration was 89 days.

Men were slightly more likely to default than women but the difference was not statistically significant (OR 1.33, 95% CI 0.79–2.25). To assess the effect of age, subjects were divided into 6 age groups: 'under 25', '25–34', '35–44', '45–54', '55–64' and '65+'. The risk of defaulting was twice higher in the '65+' age group when compared to the 'under 25' age group but the difference was not statistically significant (OR 2.03, 95% CI 0.65–6.36). HIV infection was present in 6 defaulters (5%) and in 8 controls (6%), there was no apparent association with default (OR 0.78, 95% CI 0.26 – 2.30).

Individuals who were pensioners or unemployed had a higher risk for default compared to individuals who were employed by the government, OR 4.07 (95% CI 1.57–10.52) and OR 2.73, (95% CI 1.28 – 5.86) respectively. Also individuals who abused alcohol had a higher risk for default, OR 6.01 (95% CI 1.68 – 19.47). Homelessness is probably a strong risk factor (p = 0.01), but could not be fully evaluated since in the study population there were only six homeless, all of whom defaulted (table 1). Eighty six out of 126 defaulters assessed (68%) had at least one of the 4 risk factors identified: 'Unemployed', 'Pensioner', 'Alcohol abuse' or 'Homeless'.

Smear status at the start of treatment was assessed as a potential influential factor in multivariate analysis. 'Smear positive' turned out to be a protective factor (OR 0.42, 95% CI 0.24–0.75).

The two single most commonly recorded reasons for default in the patient records were 'patient refusing further treatment' and 'violation of hospital rules'. 'Violation of hospital rules' implies that the patient was expelled from the TB hospital because he or she did not follow the rules. Twenty three defaulters (18%) interrupted treatment for this reason. Such 'violations' may range from simply leaving the hospital for some days without prior permission, to displaying aggressive behaviour. We found a strong association between 'violation of hospital rules' as a reason for default and alcohol abuse (Chi Square 15.3, p = 0.0001). Thirty three defaulters (27%) were said to have 'refused further treatment', which implies that the patient chose not to continue treatment under the conditions offered. The other frequently recorded reasons were migration (20 cases, [16%] of which 9 went abroad and 11 migrated internally) and 'side effects' (12 cases, 10%). Recorded reasons for default were comparable for the two sexes and for different age groups.

A separate finding was that 42 (33%) out of 126 defaulters did continue to take some form of treatment. They had interrupted treatment according to 'DOTS' but had continued treatment under 'Non-DOTS' conditions. In the district TB register, they were recorded as 'defaulters', however in their patient files entries were found indicating that they had continued TB treatment. They either bought tablets themselves, or were given tablets by health services staff without direct observation. Risk factors identified remained statistically significant after excluding 'Non-DOTS treatment' cases from the multivariate analysis. Since it was impossible to determine whether these patients were actually on a regular treatment regimen, we decided not exclude them from the defaulters group in our final analysis. There were no statistically significant associations between 'Non-DOTS treatment' and any of the other factors assessed.

## Discussion

Default is a serious problem in the TB program of Tashkent city and occurs mostly during the intensive phase. Pensioners, unemployed and persons abusing alcohol are at an increased risk of default, so are homeless. The fact that certain social problems tend to be more common in urban areas could provide a possible explanation for the higher default rate in Tashkent in comparison to the rest of Uzbekistan.

The patient records show various reasons for default, none of which universally apply to the bulk of defaulters. The most common reasons listed are refusal by the patient to continue treatment; and the patient being expelled from the hospital because of not adhering to the rules. Though unemployment was confirmed as a statistically significant risk factor for default, migration according to the records accounted for only 16% of all default. The initial assumption that patients default mostly because they move abroad in search of job opportunities could not be substantiated. Contrary to the findings of Jakubowiak et al in Russia [14], being smear positive at the start of treatment turned out to be a protective factor against default. Smear negative pulmonary TB may have been over diagnosed, only 394 out of 1087 pulmonary TB patients (36%) were smear positives. A TB patient wrongly diagnosed will not benefit from TB treatment and is therefore less likely to complete such treatment.

Most default in Tashkent (61%) occurs during the intensive phase when TB patients are hospitalized. Data from studies in other developing countries analyzed in a recent systematic review are inconclusive but suggest that in those countries most default occurs during the continuation phase [17].

System factors as well as patient factors appear to play a role. With 'refusal of further treatment' and 'violation of hospital rules' being the most commonly recorded reasons for default, it appears that being on admission in a TB hospital is a major obstacle. On top of this, the (hospital based) intensive phase is usually continued longer than required; this becomes apparent from the median treatment duration of 89 days for those who defaulted immediately upon its completion. A further indication of the importance of system factors in default is the fact that one third of 'defaulters' did not really default but continued treatment under 'non-DOTS' conditions.

The so-called 'non-DOTS' group is at high risk of irregular treatment and associated development of drug resistance [18]. Moreover these patients are not further evaluated. Even if treatment is no longer directly observed, it is essential that treatment regimens are appropriate and that outcomes are evaluated. Already Tashkent is experiencing high levels of primary MDR-TB, of which the 10% failure rate among new smear positives is most probably a reflection [2]. The 9% death rate might be related to late case finding, it might also be related to the MDR-TB problem. Patients being treated without being registered in the district TB register and therefore not being subject to further evaluation, is probably an indication of persistent reluctance to the DOTS strategy amongst a number of TB specialists. It also prompts us to think about the reasons why so many patients prefer 'non-DOTS' treatment, outside the regular TB control system. Since hospitalization appears to be a problem, a further shift towards ambulatory treatment may reduce default. As an added benefit this would reduce the potential for nosocomial transmission; Gelmanova et al. [12] showed prolonged admission in TB hospitals to be an important risk factor in acquiring MDR-TB. If for legal reasons hospitalization during the intensive phase cannot be avoided, intensive phase treatment should be limited to a maximum of 60 days provided sputum smear conversion has occurred.

Another weakness in the system that became apparent is the transition from intensive phase, usually as in-patient in a TB hospital, to continuation phase, usually as out-patient at a PHC facility. A substantial proportion of defaulters were lost in between these two phases. To address this problem, the referral system between TB hospital and PHC services needs to be improved. One possible solution would be to appoint for each TB patient a case manager who will be responsible for following up the patient from the moment of diagnosis until he is finally discharged from treatment.

Certain groups may require special attention; these include jobless, pensioners, homeless and alcoholics. For pensioners and unemployed, specific measures such as a system of social support including the use of incentives and enablers could be designed. A study from Brazil [9] showed that social support is highly valued by poor TB patients. For homeless and alcoholics, a flexible approach to ambulatory treatment has shown promising results in Tomsk, Russia [15].

While conducting this study we talked to health services staff and studied patient records but we did not talk to the patients themselves. An advantage of this approach was that we were able to obtain information on almost all defaulters, thus excluding a potential selection bias mentioned in several earlier studies [7-9]. The study design enabled us to quickly obtain a general picture of defaulters in Tashkent; it allowed us to identify who the defaulters are and when they default, but it did not allow us to fully identify the reasons why patients default. With 45% of default being due to 'refusal of further treatment' and 'violation of hospital rules', there is a need to further investigate why so many patients are either not satisfied with or not able to comply with the conditions under which they are treated. A qualitative follow-up study in which both patients and health workers are interviewed might provide the answer to this question [19].

## Conclusion

Default in Tashkent City occurs mostly during the intensive phase while patients are hospitalized. A number of risk groups have been identified that require special attention; these include unemployed, pensioners, homeless and alcoholics.

Obligatory in-patient treatment during the intensive phase and a weak system of transfer to general PHC facilities upon completion of the intensive phase, are important system factors leading to default. Default can be substantially reduced by shortening the duration of in-patient treatment and improving the referral system between TB in-patient facilities and PHC services.

## Competing interests

The authors declare that they have no competing interests.

## Authors' contributions

EH: Protocol design, data analysis, first author. MK: Support protocol design, coordinate data collection, support data analysis, review of manuscript. SU: Data collection, support data analysis, review of manuscript. UA: Data collection, support data analysis, review of manuscript. UY: Data collection, support data analysis, review of manuscript. MJvdW: Support data analysis, review of manuscript. GU: Review of manuscript. JV: Review of manuscript.

## Pre-publication history

The pre-publication history for this paper can be accessed here:


